# Versatile genetic assembly system (VEGAS) to assemble pathways for expression in *S. cerevisiae*

**DOI:** 10.1093/nar/gkv466

**Published:** 2015-05-08

**Authors:** Leslie A. Mitchell, James Chuang, Neta Agmon, Chachrit Khunsriraksakul, Nick A. Phillips, Yizhi Cai, David M. Truong, Ashan Veerakumar, Yuxuan Wang, María Mayorga, Paul Blomquist, Praneeth Sadda, Joshua Trueheart, Jef D. Boeke

**Affiliations:** 1 Department of Biochemistry and Molecular Pharmacology, New York University Langone School of Medicine, New York City, NY 10016, USA; 2 Institute for Systems Genetics, New York University Langone School of Medicine, New York City, NY 10016, USA; 3 High Throughput Biology Center, Johns Hopkins University School of Medicine, Baltimore, MD 21205, USA; 4 Department of Biomedical Engineering and Institute of Genetic Medicine, Whiting School of Engineering, Johns Hopkins University, Baltimore, MD 21218, USA; 5 DSM Nutritional Products, Lexington, MA 02421, USA

## Abstract

We have developed a method for assembling genetic pathways for expression in *Saccharomyces cerevisiae*. Our pathway assembly method, called VEGAS (Versatile genetic assembly system), exploits the native capacity of *S. cerevisiae* to perform homologous recombination and efficiently join sequences with terminal homology. In the VEGAS workflow, terminal homology between adjacent pathway genes and the assembly vector is encoded by ‘VEGAS adapter’ (VA) sequences, which are orthogonal in sequence with respect to the yeast genome. Prior to pathway assembly by VEGAS in *S. cerevisiae*, each gene is assigned an appropriate pair of VAs and assembled using a previously described technique called yeast Golden Gate (yGG). Here we describe the application of yGG specifically to building transcription units for VEGAS assembly as well as the VEGAS methodology. We demonstrate the assembly of four-, five- and six-gene pathways by VEGAS to generate *S. cerevisiae* cells synthesizing β-carotene and violacein. Moreover, we demonstrate the capacity of yGG coupled to VEGAS for combinatorial assembly.

## INTRODUCTION

The production of high-value metabolites in microorganisms suited to industrial-scale growth can overcome costly issues associated with traditional production routes, including yield, extraction or complicated synthesis procedures. To achieve this, the biosynthetic pathway of interest must be re-constructed in an appropriate host organism, typically chosen because it is well characterized and genetically tractable. *Saccharomyces cerevisiae* is a favored eukaryotic microorganism for metabolic engineering because it is industrially robust, generally regarded as safe and highly amenable to and tolerant of genetic manipulation. Many recent successes in the metabolic engineering of *S. cerevisiae* have been described (reviewed in (1)), most notably the cost-effective production of artemisinic acid, a precursor to the anti-malarial drug artemisinin (2). Engineering of the host genome to redirect endogenous pathways and optimizing the expression levels of non-native biosynthetic genes are key to successful metabolic engineering projects.

Here we focus on the challenge of assembling biosynthetic pathways for expression in *S. cerevisiae*, guided by the synthetic biology principles of modular workflows using standardized parts. Existing DNA assembly strategies can be divided into two main classes, each with notable advantages. The first class, ‘overlap-directed assembly’ methods such as Gibson isothermal assembly (3) and assembly directly in yeast (4,5) leverage terminal sequence homology and enzymes for resection and DNA repair to assemble specified, adjacent parts. These types of assembly methods are sequence independent since no specific sequences (i.e. enzyme recognition sequences) are required to be present. Overlap-directed assembly methods provide flexibility as order and orientation of parts may be changed on the fly, although this typically requires new primer sets and PCR, which can introduce mutations. In contrast, the second DNA assembly class depends on specific restriction enzymes and their corresponding recognition sequences, either pre-existing or designed, in the DNA fragments to be assembled. Golden Gate assembly (6,7) uses the activity of type IIS restriction enzymes, such as BsaI or BsmBI, which cut outside of their recognition sequences to expose designer overhangs, enabling directional and seamless assembly of parts. Golden Gate embodies the concept of modular assembly, especially when applied to genes within genetic pathways, as each unit may be decomposed into promoter (PRO) and terminator (TER) regulatory elements that flank coding sequences (CDSs).

Here we present a Versatile genetic assembly system (VEGAS), which exploits the advantageous features of both classes of DNA assembly systems, providing a simple, new method to construct genetic pathways for expression in *S. cerevisiae*. We assemble yeast genes, or transcription units (TUs), using a standardized version of Golden Gate that we call yeast Golden Gate (yGG) (8). In the yGG reaction, each TU is assigned a pair of VEGAS adapters (VAs) that assemble up- and downstream of each TU; it is the VA sequences that subsequently provide terminal homology for overlap-directed assembly by homologous recombination ‘*in yeasto*’. We apply the VEGAS methodology to the assembly of the β-carotene and violacein biosynthetic pathways, whose pigmented products are visible in yeast colonies. Moreover, we demonstrate the capacity of VEGAS for combinatorial assembly.

## MATERIALS AND METHODS

### Design of VA sequences

From a previously generated, in-house collection of 10-mer sequences that rarely occur in the *S. cerevisiae* genome, 60 mers were randomly produced by concatenation *in silico*. The eighteen 60 mers with the lowest similarity to the *S. cerevisiae* genome were selected to comprise the initial set of VA sequences reported here. For cost minimization, the VA sequences were subsequently shortened to 57 mers by deleting three terminal base pairs (Table 1). Alternatively, the web-based tool R2oDNA can be used to design orthogonal sequences (9).

**Table 1.** VEGAS adapter sequences

Name	Sequence (5′ - 3′)
VA1^a^	CCCCTTAGGTTGCAAATGCTCCGTCGACGGGATCTGTCCTTCTCTGCCGGCGATCGT
VA2^b^	TGACGCTTGGATGCGTGACCCCGTACGTCATGACCCGTCATGGGTATGTAAGCGAAG
VA3	GGAGGTACTGGCCTAGCGTCGTGGCCCGGGAGAGACAGTTTAGTAGTGACTCGCGGC
VA4	TTGGCGTTAATTGTAGCTTATTTCCCGCCCTGTGATTGAGGCGGGATGGTGTCCCCA
VA5	GACTAAGACTCTGGTCACGGTTCAGAAGTGGACGATGCATGTCGTCGGGCTGATAGA
VA6	TGCACGGCGCTAGGTGTGATATCGTACACTTGGGAGAAGTCAGATACGATTGCGGCT
VA7	TAGCGGCGCCGGGAAATCCAGCATATTCTCGCGGCCCTGAGCAGTAGGTGTCTCGGG
VA8	GAGTCTACGTTACACCTGAACTCGCATGTCTGGGGTTGTGGTCAGGCCTTGTCAATT
VA9	GCGTACTGGCCGCCCGGGCCTGATGTGGCCGTCCTATTAGCATTGTACACCCTCATT
VA10	CTTGAATCGGCTTTAGGATCCGGTACTGCCGACGCACTTTAGAACGGCCACCGTCCT
VA11	GCAAGTTTTGAAGAGGTGTAAACTCTCCGCAGCACCTCCGGACTATGCCCGAGTGGT
VA12	TGAAGCTACGCGCCGAGCGTCTGACTCCTTTAGTCCGCGTCATCGCTTTGAGCGCGT
VA13	TCCGGATCCCTTTCGGTCCATATAGCGGATTTCCATAGACGTAGACCGCGCCAATGT
VA14	GACGACGCGTTCTGTGTCTTCGTTGCGGCTCTGCGCTTGGTCGTTGGCGACGGCCGT
VA15	TGTAAGGGCGTCTGTTAACCCAAGGTCCCTCGAACCGTATGCAGAGCCGTGGCTACG
VA16	TATCGCGGGTGCGTGCATCGACAAGCCATGCCCACCTTCTGGTCGATTGGGCTGGCG
VA17	CATCCATCGATATTTGGCACTGGACCTCAACGCTAGTGTTCGCGGACTGCACTACCT
VA18	GATTAAGGGGCATACCGTGCCTATCCTGGTAATTGTGTAGGCTACCTGTCTGTATAC

^a^Encoded terminally on the left arm of the linearized VEGAS assembly vector.

^b^Encoded terminally on the right arm of the linearized VEGAS assembly vector.

### Vector construction

To construct the yGG acceptor vector for TUs destined for VEGAS, pUC19 (10) was modified using a previously described method (11). Briefly, all pre-existing instances of BsaI and BsmBI sites were re-coded or deleted and a custom multiple cloning site (MCS) was installed, encoding an *E. coli* RFP expression cassette flanked by outward-facing BsaI sites designed to leave 5′ and 3′ VA designer overhangs (top strand: CCTG and AACT, respectively). Additionally, neighboring NotI and FseI sites, or inward-facing BsmBI sites were further encoded outside of the BsaI sites to facilitate excision of assembled VA-flanked TUs from the construct. Plasmid identification numbers are pNA0178 (NotI and FseI) and pJC120 (BsmBI). To construct the VEGAS assembly vector, a previously constructed yGG acceptor vector (11), pAV116, which derives from pRS416 (12), was used. VA1 and VA2 sequences plus BsaI sites (as shown in Figure 2) were then introduced up- and downstream of an *E. coli* RFP expression cassette.

**Figure 1.**
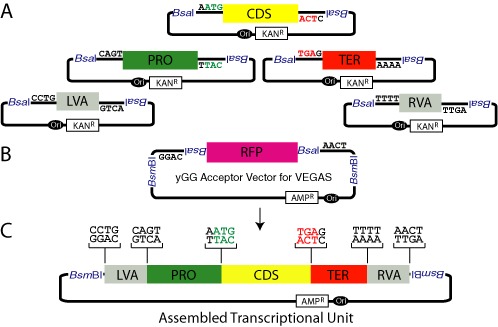
Yeast Golden Gate (yGG) to assemble transcription units (TUs) flanked by VEGAS adapters. (**A**) yGG reactions to build TUs destined for VEGAS pathway assembly in *S. cerevisiae* include five parts: a left VEGAS adapter (LVA), a promoter (PRO), a coding sequence (CDS), a terminator (TER) and a right VEGAS adapter (RVA). Each part is flanked by inwardly facing recognition sequences for the BsaI restriction enzyme, an ‘offset cutter’ which cuts outside its recognition sequence (at positions 1/5 bp downstream) to expose the indicated four base-pair overhangs. All parts are cloned into vectors encoding kanamycin resistance (KAN^R^) and an *E. coli* replication origin (Ori). (**B**) The yGG acceptor vector for VEGAS is designed such that outwardly facing BsaI sites expose overhangs corresponding to the 5′ LVA and 3′ RVA overhangs to promote assembly of the TU in the vector during a one-pot restriction-digestion reaction. The RFP cassette, built for expression in *E. coli*, is cut out of the vector when a TU correctly assembles, enabling white–red screening. The yGG acceptor vector encodes resistance to ampicillin (AMP^R^) (**C**) The structure of a VA-flanked TU assembled by yGG. An assembled TU plus the flanking VA sequences may be released from the yGG acceptor vector by digestion with BsmBI.

**Figure 2.**
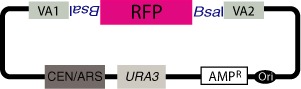
VEGAS vector for pathway assembly. Digestion with BsaI linearizes the VEGAS assembly vector, releasing an RFP cassette and exposing terminal VA sequences VA1 and VA2 on the vector arms. Assembly of a genetic pathway by homologous recombination in yeast is selected on medium lacking uracil based on expression of *URA3* from the vector backbone and mitotic stability in dividing yeast cells ensured based on the centromere (CEN) and autonomously replicating sequence (ARS) combination encoded on the vector. The VEGAS assembly vector also encodes resistance to ampicillin (AMP^R^) plus an *E. coli* replication origin (Ori); assembled constructs can therefore be recovered from yeast into *E. coli*.

### Parts cloning

The β-carotene CDS parts *crtE, crtI* and *crtYB*, were amplified from genomic DNA of an *S. cerevisiae* strain previously engineered to express the pathway (13). Codon optimized violacein biosynthetic enzyme CDS parts, *vioA, vioB, vioC, vioD and vioE*, were synthesized. The truncated version of *HMG1* (*tHMG1*) plus all PRO and TER parts were amplified from genomic DNA extracted from the BY4741 (14) strain of *S. cerevisiae*. Primers used for amplification included overhangs encoding inward-facing BsaI sites separated by one base from the appropriate yGG-compatible overhangs. All parts were subcloned using the Zero Blunt TOPO PCR cloning kit (Life Technologies; 45–0245), transformed into *E. coli* (Top10 cells) and sequence verified. CDS parts that encoded BsaI or BsmBI sites were re-coded by Multichange Isothermal mutagenesis as previously described (11). All parts and their corresponding sequence files are available upon request.

### yGG into the VEGAS yGG acceptor vector

100 ng of yGG acceptor vector (pJC120 for all experiments described in this work) plus equimolar amounts of each part for assembly (LVA, PRO, CDS, TER, RVA) were combined in a Golden Gate reaction consisting of 1.5 μl 10X T4 DNA ligase reaction buffer (New England Biolabs, M0202), 0.15 μl 100X Bovine Serum Albumin (BSA, New England Biolabs), 600U T4 DNA ligase (rapid) (Enzymatics, L6030-HC-L) and 10U of BsaI (New England Biolabs, R0535) in a final volume of 15 μl. One-pot digestion-ligation assembly was carried out in a thermocycler by performing 25 cycles of [37°C 3 min, 16°C 4 min], followed by 50°C 5 min, and 80°C 5 min. We have also described several modifications to improve the efficiency of yGG (8). For ‘terminal homology VEGAS’ experiments, 5 μl of each yGG reaction was transformed into Top10 *E. coli* and plated on LB plates supplemented with carbenicilllin (75 μg/ml). White colonies were selected for verification of assembly constructs by restriction digest. For combinatorial assembly, PRO or TER parts were mixed in equal molar amounts prior to yGG assembly.

### Terminal homology VEGAS

∼1 μg of yGG-assembled, VA-flanked TU constructs were digested with BsmBI (New England Biolabs, R0580) in a final volume of 20 μl. 2 μl (∼100 ng) of each digestion product was used directly for yeast transformation along with ∼50 ng of BsaI-linearized VEGAS assembly vector (pJC170 for all experiments described in this work). Yeast transformations were carried out as previously described (15) except cells were heat shocked for only 15 min in the presence of 10% DMSO at 37°C and prior to plating were incubated in 400 μl of 5 mM CaCl_2_ for 10 min at room temperature. For all VEGAS yeast transformations, following primary selection on SC–Ura plates (incubated 3 days at 30°C), plate images were taken and transformation plates were replica plated onto YPD medium supplemented with G418 (200 μg/ml). A second set of plate images was taken three days post-replica plating.

### PCR-mediated VEGAS

Primers were designed to anneal to the leftmost and rightmost ends of the LVA and RVA sequences, respectively. Each primer additionally encoded 30 bp of overhang sequence homologous to the adjacent VA sequence. 1 μl of each yGG reaction was used directly in a PCR reaction with Phusion High-Fidelity DNA Polymerase (M0530L) to amplify the VA-flanked TU and incorporate neighboring homology. 5 μl of each PCR reaction was transformed directly into yeast along with ∼50 ng of BsaI-linearized VEGAS assembly vector (pJC170 for all experiments described in this work). Yeast transformation and replica plating steps were performed as described in the ‘Terminal Homology VEGAS’ section.

### Plasmid recovery from yeast

Following VEGAS, assembled constructs encoding the β-carotene and violacein pathways were recovered from yeast as previously described (5) except that in all cases constructs were recovered from 3 ml of cultured yeast (SC–Ura), inoculated from a single yeast colony, and the blue–white *E. coli* screening step following transformation was omitted. For combinatorial assembly of the β-carotene pathway, PRO and TER parts flanking each CDS were determined by Sanger sequencing of the recovered plasmid.

### Carotenoid production

Four constructs encoding β-carotene pathways (pJC175, orange; pJC178, bright yellow; pJC181, pink; pJC184, light yellow), each a product of combinatorial PCR-mediated VEGAS (Figure 4E–H), were used. Three independent colonies of each were inoculated into 10 ml of YPD medium supplemented with G418 (200 μg/ml) and grown to saturation (3 days at 30°C, 250 rpm). Carotenoids were extracted using a PRECELLYS^®^ 24 high-throughput tissue homogenizer. Briefly, 1 ml of culture was pelleted in a PRECELLYS tube and the pellet was extracted with 1 ml tetrahydrofuran (containing 0.01% butylhydroxytoluene (BHT)) by homogenization for 3 × 15 s at 6500 rpm. Following centrifugation for 5 min at 4°C, 800 μl was then transferred to a glass vial. Extracts were dried down and resuspended in 80 μl dichloromethane followed by 720 μl of a 50:50 (v/v) mixture of heptane and ethyl acetate (containing 0.01% BHT). HPLC analysis of carotenoids was performed essentially as described (16).

**Figure 3.**
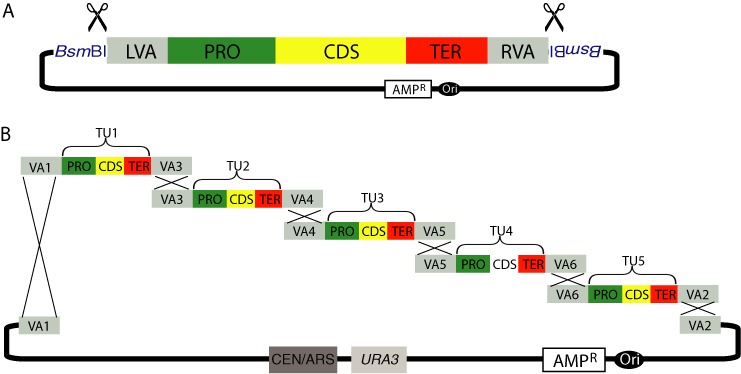
VEGAS with adapter homology to assemble a five-gene pathway. (**A**) The pathway consisting of VA-flanked TUs assembled by yGG may be released in one piece from the yGG acceptor vector by digestion with BsmBI (scissors). (**B**) A genetic pathway may be assembled into the linearized VEGAS assembly vector in *S. cerevisiae* by homologous recombination between VAs that flank TUs (TU1–5). X's indicate homologous recombination.

**Figure 4.**
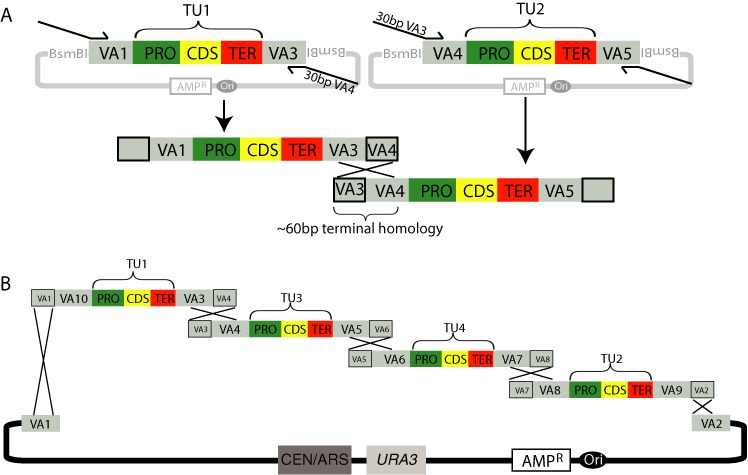
VEGAS with PCR-mediated homology to assemble a four-gene pathway. (**A**) TUs flanked by unique VAs are assembled by yGG and then subjected to PCR using primers that introduce terminal homology between adjacent parts. In this example, the reverse primer amplifying TU1 encodes 30 bp of sequence homology to VA4 and the forward primer amplifying TU2 encodes 30 bp of sequence homology to VA3. Together this generates 60 bp of terminal sequence homology between TU1 and TU2 for the homologous recombination machinery in *S. cerevisiae* to assemble a linear piece of DNA *in vivo*. (**B**) Gene order may be changed by using different overhang primers; here the final pathway structure becomes TU1–TU3–TU4–TU2, although any order and/or gene orientation is possible and depends only on primer design.

## RESULTS

### yGG for VEGAS

#### yGG to assemble TUs destined for VEGAS

The yGG method (8) defines genes as TUs composed of three functionally distinct types of parts: PROs (these parts subsume UAS, promoter and 5′ UTR sequences as a single part), CDSs and TERs (consisting of 3′ UTR and polyadenylation signals). In brief, yGG exploits type IIS restriction enzymes that cut outside of their recognition sequences exposing designer, ‘biologically meaningful’ overhangs to promote assembly of functional TUs (PRO-CDS-TER) in specially constructed acceptor vectors. The critical distinction made for TUs destined for VEGAS pathway assembly is the addition of two additional VA parts into the assembly. Here, one VA is designed to assemble upstream of the PRO (LVA: left VEGAS adapter) and the other for assembly downstream of the TER (RVA: right VEGAS adapter). The yGG reaction with VA parts thus generates the following structure: (vector end)-LVA-PRO-CDS-TER-RVA-(other vector end) (Figure 1). The RVA and LVA designer overhangs and acceptor vector built specifically for assembling VA-flanked TUs are described below. Importantly, yGG can be carried out in a ‘one-pot reaction’, is compatible with combinatorial assembly (i.e. pools of PROs and TERs in a single yGG reaction) and is amenable to automation.

#### Designer overhangs

We previously defined biologically relevant yGG overhangs for PRO, CDS, and TER parts (Figure 1) that are highly compatible with gene expression (8). To enable VEGAS, we further define overhang sequences that enable assembly of a VA upstream of the PRO (LVA) and a second VA downstream of the TER (RVA). The overhangs for the LVA part are CCTG-**LVA**-CAGT and the overhangs for the RVA part are TTTT-**RVA**-AACT. The complete structure of a VA-flanked TU assembled by yGG for VEGAS is as follows: (vector end)-CCTG-**LVA**-CAGT-**PRO**-AATG -**CDS**-TGAG-**TER**-TTTT-**RVA**-AACT (other vector end) (Figure 1). For clarity, the underlined bold letters represent the parts.

#### VEGAS adapters

VAs are designed to be orthogonal in sequence with respect to the native *S. cerevisiae* genome (see Materials and Methods section). For compatibility with yGG assembly, each VA is subcloned into a kanamycin-resistance vector flanked by inward-facing BsaI sites; digestion with BsaI exposes overhangs encoded for yGG assembly. Each VA sequence (Table 1) is subcloned with yGG overhangs for assembly into either the LVA (CCTG-**LVA**-CAGT) or RVA (TTTT-**RVA**-AACT) position. As a result, each VA sequence can be assigned for assembly into either the LVA or RVA TU position in any yGG reaction. Our collection of VA sequences (Table 1) currently contains 18 unique VA sequences, each 57 bases in length (Table 1). The VA collection can easily be expanded by designing new orthogonal sequences. The two main considerations for designing additional VA sequences include: (i) the sequence must not contain BsaI or BsmBI sites (or any other type IIS restriction sites that may be used for TU assembly) or sites for enzymes used subsequently to release the assembled TU from the yGG acceptor vector (e.g. FseI or NotI); and (ii) the sequence must be distinct from the *S. cerevisiae* genome.

#### yGG acceptor vector designed for assembling VA-flanked TUs

We have constructed acceptor vectors with a custom MCS for assembly of VA-flanked TUs. These vectors derive from pUC19 (10), with all pre-existing instances of BsaI and BsmBI restriction sites removed to support the function of the newly installed, custom MCS, which is dependent on the sequential action of these two enzymes. In detail, the MCS encodes an RFP cassette with an *E. coli* PRO and TER sequences that confer a red colony color upon introduction into *E. coli*. The RFP cassette is flanked by outwardly facing BsaI sites that expose the required VA overhangs for yGG assembly (LVA 5′ end: CCTG; RVA 3′ end: AACT). Successful yGG assembly cuts the RFP cassette out of the plasmid allowing identification of positive clones by white/red screening. Finally, beyond each BsaI site is encoded an inward facing BsmBI site that can be used to release assembled TUs for subsequent VEGAS assembly. For assemblies that are incompatible with BsmBI digestion to release the assembled TU (for example if any of the parts encode an internal BsmBI site), we have also built additional vectors that use NotI or FseI, two rare cutters with 8bp recognition sequences, to release assembled TUs flanked by VAs. In principle any enzyme that does not cut internally to the assembly VA-flanked TU can be built into this yGG acceptor vector.

### VEGAS to assemble pathways for expression in yeast

The VAs flanking each assembled TU are a key requirement for VEGAS. Specifically, each VA provides 57 bp of unique sequence that can be leveraged for homologous recombination-dependent pathway assembly *in vivo* into a specially designed VEGAS acceptor vector (Figure 2). Importantly, this approach supports modularity during assembly and re-usability of parts, thereby allowing combinatorial assembly of TUs. We have developed two distinct VEGAS workflows that are described below. Briefly, in the first instance the VAs themselves provide terminal sequence homology for pathway assembly (Figure 3), while in the second instance the VAs serve as primer binding sites for overhang extension PCR to generate terminal homology (Figure 4). The latter workflow has the added advantage that the order and orientation of genes in the pathway can be changed even after TU assembly simply by designing new sets of primers. In both cases, a common VEGAS vector is used for pathway assembly.

#### VEGAS vector

A VEGAS vector (Figure 2) is used for pathway assembly by homologous recombination in *S. cerevisiae*. It encodes all sequences required for mitotic stability in yeast, including a centromere (CEN), replication origin (autonomously replicating sequence (ARS)) and a selectable marker. A 2 μm origin can also be used in place of the CEN/ARS combination. Because the final assembled construct in yeast is circular, there is no requirement for telomeres. Further, the vector encodes a selectable marker and replication origin for propagation in *E. coli*. Our VEGAS assembly vector design includes a custom MCS in which an *E. coli* RFP expression cassette is flanked by outward facing BsaI sites; all other instances of BsaI sites have been recoded or removed from the vector. Digestion with BsaI linearizes the vector, releasing the RFP cassette and exposing previously incorporated terminal VA sequences (VA1 and VA2).

#### VA homology VEGAS

In the first VEGAS workflow, the order and orientation of all pathway genes is defined at the outset of the experiment and VAs are assigned to each TU based on the selected position. Specifically, the LVA assigned to the left-most positioned TU must encode VA sequence ‘1’ (VA1, Table 1) to match one end of the linearized VEGAS assembly vector (see above); adjacent TUs must encode identical VA sequences assembled in the RVA and LVA positions; finally the RVA of the right-most TU must encode VA sequence ‘2’ (VA2, Table 1) to match the other end of the linearized VEGAS assembly vector (Figure 2). The TUs of the pathway of interest are assembled in individual yGG reactions, and following *E. coli* transformation and isolation of a correctly assembled construct (white colony), the VA-flanked TU inserts can be released by *Bsm*BI digestion (Figure 3A). The digestion products corresponding to all pathways TUs are then transformed into yeast along with the linearized VEGAS assembly vector and the pathway assembled by homologous recombination (Figure 3B). In this scenario, gene order and orientation in the assembled pathway are fixed once the yGG reactions are performed. The position of TUs with respect to one another can only be changed if the TUs are reassembled by yGG with newly assigned VAs.

#### PCR-mediated VEGAS

In this workflow, a unique VA sequence (Table 1) is assigned to the LVA and RVA positions of each TU in the genetic pathway. As a result, the yGG-assembled VA-flanked TUs encode no terminal sequence homology with one another or with the VEGAS assembly vector. Rather, each assembled TU is subjected to PCR amplification using primers that anneal to the VAs and encode specific overhangs that generate terminal sequence homology between adjacent TUs (and the vector). The major advantage to this workflow is the capability to change the gene order and orientation without having to rebuild each TU, as described above.

### Proof-of-concept: yGG and VEGAS to assemble the β-carotene and violacein pathways in *S. cerevisiae*

The four gene β-carotene pathway and the five gene violacein pathway serve as useful tools to develop DNA assembly strategies as pathway expression can be tracked by the development of colored yeast. Expression of violacein pathway genes (*vioA, vioB, vioC, vioD and vioE* from *Chromobacterium violaceum* (17)) can turn yeast purple (18), while expressing genes of the β-carotene pathway (*crtE, crtI, crtYB* from *Xanthophyllomyces dendrohous*) yields orange colonies (13). Color production in both cases is quantitatively and qualitatively dependent on pathway flux and thus on the expression levels of pathway genes (13,18,19). For instance, overexpression of the catalytic domain of the *S. cerevisiae* HMG CoA reductase *HMG1* (*tHMG1*) can dramatically alter carotenoid production, yielding yellow colonies (13,20). As proof-of-concept of the VEGAS methodology we have assembled carotenoid and violacein pathways for expression in *S. cerevisiae* using yGG-assembled VA-flanked TUs.

#### VA homology

**Figure 5.**
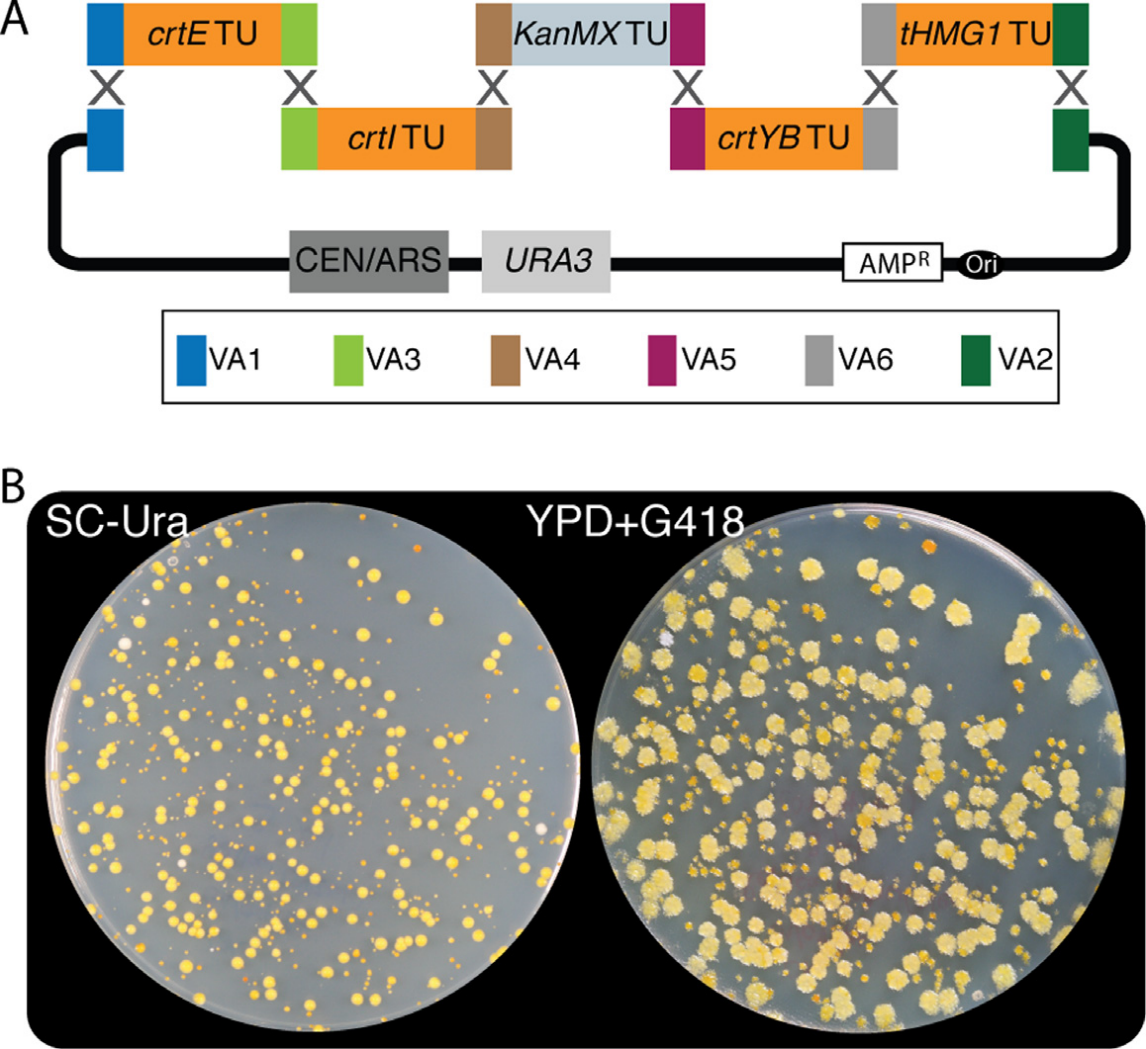
VEGAS with adapter homology to assemble the carotenoid pathway in *S. cerevisiae*. (**A**) The four β-carotene pathway genes (*crtE, crtI, crtYB* and *tHMG1*), assembled as TUs flanked by the indicated VAs (see Table 2 for PRO and TER parts), were released from the yGG acceptor vector with BsmBI digestion and co-transformed into yeast with the linearized VEGAS assembly vector. (**B**) *S. cerevisiae* colonies encoding assembled pathways develop a bright yellow color on medium lacking uracil (SC–Ura; left panel) as well as on YPD medium supplemented with G418 (right panel).

To demonstrate VEGAS using terminal homology encoded in the VA sequences, we assigned each β-carotene pathway CDS a unique *S. cerevisiae* PRO and TER (Table 2) and pre-determined the desired, left-to-right assembly order (Figure 5A). A strong PRO was assigned to each CDS for high expression of each gene. In the VEGAS experiments presented here we included the KanMX TU (pre-assembled as a PRO-CDS-TER yGG part), whose protein product yields resistance to the drug G418, to permit a secondary plate-based screening approach using an unselected marker to test for efficiency of correct assemblies in yeast. Based on the pre-defined gene assembly order (Figure 5A), we assigned the appropriate LVA and RVA to each TU. Subsequent to yGG, a correctly assembled TU (white colony) for each of the five reactions was selected and the pathway assembled by VEGAS via co-transformation of BsmBI-digested TUs plus the linearized VEGAS assembly vector. The primary selection for assembly was carried out on medium lacking uracil (SC–Ura), as the *URA3* gene was encoded on the assembly vector (Figure 5A). Almost all colonies growing on the SC–Ura plate were yellow in color, consistent with assembly of a functional pathway that includes *tHMG1* (13) (Figure 5B, left panel). Moreover, following replica plating onto YPD medium supplemented with G418, virtually 100% of colonies were G418 resistant as expected for 100% correct assembly (Figure 5B, right panel). The variation in color (light yellow versus darker yellow or even orange) between colonies may result from stochasticity in expression of pathway genes between colonies, mis-assembly (for instance the absence of *tHMG1* TU, see below), or variation in plasmid copy number (e.g. two copies versus one); indeed the yellow colony color typically normalizes across the plate with incubation for several more days.

**Table 2.** yGG parts for adapter homology-mediated assembly of the β-carotene pathway by VEGAS

TU order (left to right)	LVA	PRO	CDS	TER	RVA
1	VA1	*pTDH3*	*crtE*	*ttACS2*	VA3
2	VA3	*pPGK1*	*crtI*	*ttENO2*	VA4
3	VA4		*KanMX TU*		VA5
4	VA5	*pACT1*	*crtYB*	*ttASC1*	VA6
5	VA6	*pRPS2*	*tHMG1*	*ttCIT1*	VA2

#### PCR-mediated homology

**Figure 6.**
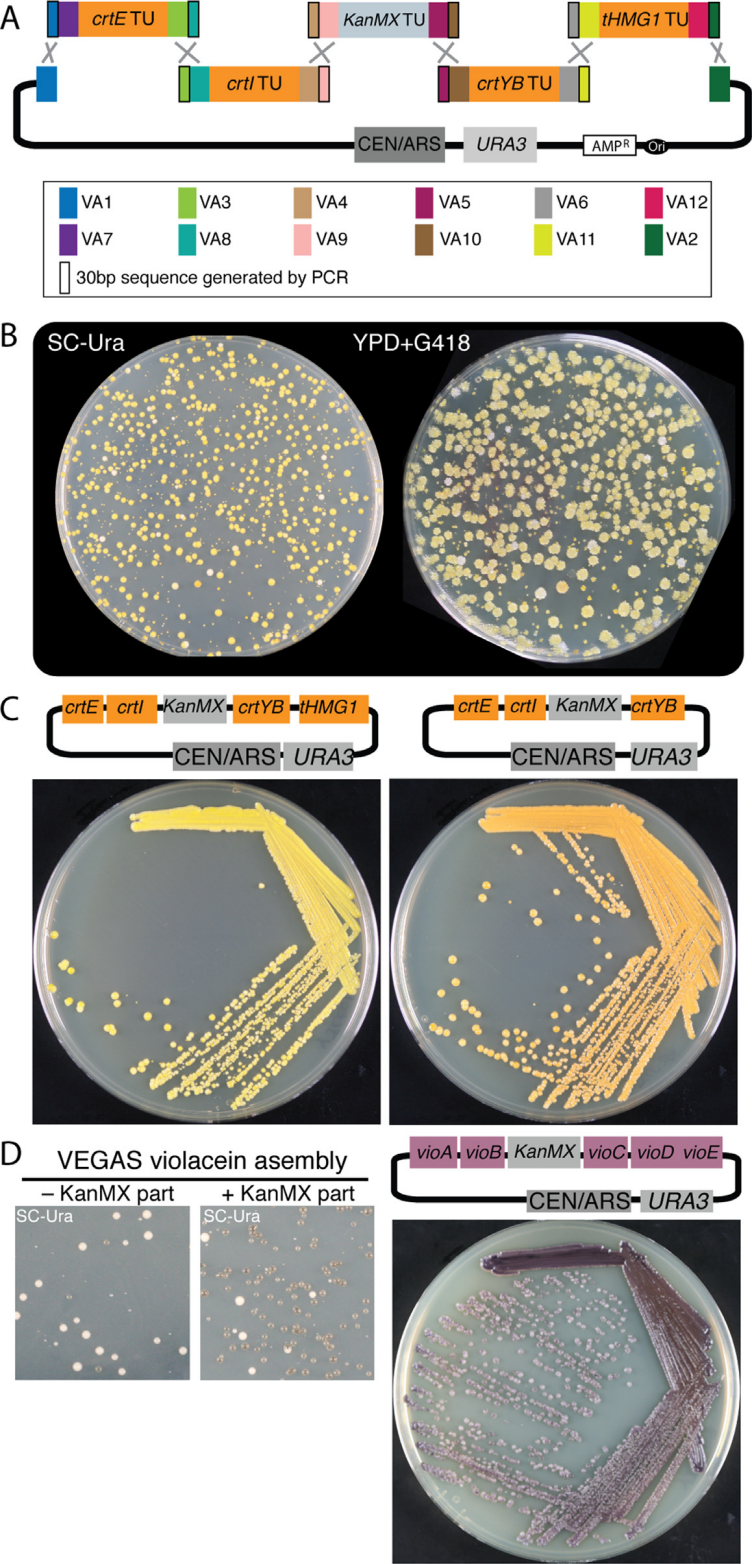
VEGAS with PCR-mediated homology to assemble the β-carotene and violacein pathways in *S. cerevisiae*. (**A**) The four β-carotene pathway genes (*crtE, crtI, crtYB* and *tHMG1*), assembled as TUs flanked by the indicated VAs (see Table 3 for PRO and TER parts), were subjected to PCR using primers to generate adjacent terminal homology between TUs and the VEGAS assembly vector. (**B**) *S. cerevisiae* colonies encoding assembled pathways develop a bright yellow color on medium lacking uracil (SC–Ura; left panel) as well as on YPD medium supplemented with G418 (right panel). (**C**) Re-streaked single colonies from three VEGAS assembly experiments. Left panel: a single yellow colony from the VEGAS assembly experiment in (B) was re-streaked for single colonies. Right panel: by designing a few new primers, a second version of the carotenoid pathway was assembled omitting the *tHMG1* TU, generating orange yeast colonies. (**D**) The violacein pathway assembled in *S. cerevisiae* yields purple colonies.

In this workflow, unique VA sequences were assigned to the LVA and RVA position for each of the four β-carotene pathway TUs plus the KanMX TU (Table 3). The PRO and TER parts for each CDS as well as the defined left-to-right gene order were not changed as compared to the adapter homology experiment described previously (Table 2 compared to Table 3). Following yGG assembly, the reaction mixtures were used directly for five independent PCRs to amplify each TU with primers encoding ∼20 nucleotides (nt) of sequence to anneal to the VA plus ∼30 nt of assigned neighboring homology sequence; together this yielded ∼50 bp of terminal homology between adjacent parts for VEGAS (Figure 6A). The PCR products were co-transformed along with the linearized VEGAS assembly vector into yeast and selection for assembly was carried out on SC–Ura medium. Here ∼95% of colonies developed a yellow color on SC–Ura and virtually 100% of colonies were also G418 resistant (Figure 6B). Compared to the adapter-mediated homology assembly (Figure 5B), more colonies appeared white in color (∼5% compared to ∼1% in Figure 5B) and most of these were also G418 resistant, suggesting a slightly lower fidelity of assembly in this approach. When a single yellow-colored, Ura^+^, G418^r^ colony was restreaked on YPD medium supplemented with G418, all resulting colonies were of a uniform yellow color (Figure 6C, left panel). The assembled pathway from this colony was recovered into *E. coli* and the plasmid structure confirmed by digestion and sequencing (data not shown).

**Table 3.** yGG parts for PCR-mediated assembly of the β-carotene pathway by VEGAS

TU order (left to right)	LVA	PRO	CDS	TER	RVA
1	VA7	*pTDH3*	*crtE*	*ttACS2*	VA3
2	VA8	*pPGK1*	*CrtI*	*ttENO2*	VA4
3	VA9		*KanMX TU*		VA5
4	VA10	*pACT1*	*crtYB*	*ttASC1*	VA6
5	VA11	*pRPS2*	*tHMG1*	*ttCIT1*	VA12

To demonstrate versatility of the PCR-mediated VEGAS approach, we assembled a different version of the β-carotene pathway, this time omitting the *tHMG1* TU. To accomplish this, we re-used the previously yGG assembled, VA-flanked TUs for *crtE, crtI, KanMX* marker and *crtYB*, and simply amplified the crtYB TU with a different primer encoding terminal homology to the VEGAS (Figure 6C, right panel). Transformation plates resembled those shown in Figure 6B but the assembly yielded colonies producing an orange color (Figure 6C, right panel). The structure of assemblies producing orange yeast cells was validated by recovery into *E. coli* and digestion (data not shown).

To push the assembly limit of VEGAS we next constructed the violacein pathway using PCR-mediated VEGAS of the five violacein TUs plus the KanMX cassette; together this was a seven-piece assembly including the vector backbone. TUs were assembled with flanking VAs by yGG (Table 4), and terminal homology between adjacent parts was introduced by PCR. Transformation into yeast of all parts required for pathway assembly, as compared to a control experiment omitting the KanMX part, yielded a substantial increase in the number of colonies producing a purple pigment on the primary SC–Ura transformation plates (Figure 6D). This color developed in all colonies upon re-streaking (Figure 6D). White colonies may arise from mis-assemblies or from circularization of the parental, empty VEGAS vector. The structure of assemblies producing purple yeast colonies was validated by recovery into *E. coli* and digestion and found to be 100% (7/7 independent colonies, data not shown).

**Table 4.** yGG parts for PCR-mediated assembly of the violacein pathway by VEGAS

TU order (left to right)	LVA	PRO	CDS	TER	RVA
1	VA7	*pTDH3*	*vioA*	*ttACS2*	VA3
2	VA8	*pPGK1*	*vioB*	*ttENO2*	VA4
3	VA9		*KanMX TU*		VA5
4	VA10	*pACT1*	*vioC*	*ttASC1*	V6
5	VA11	*pRPS2*	*vioD*	*ttCIT1*	VA12
6	VA16	*pZEO1*	*vioE*	*ttFUM1*	VA5

### yGG and VEGAS for combinatorial pathway assembly

**Figure 7.**
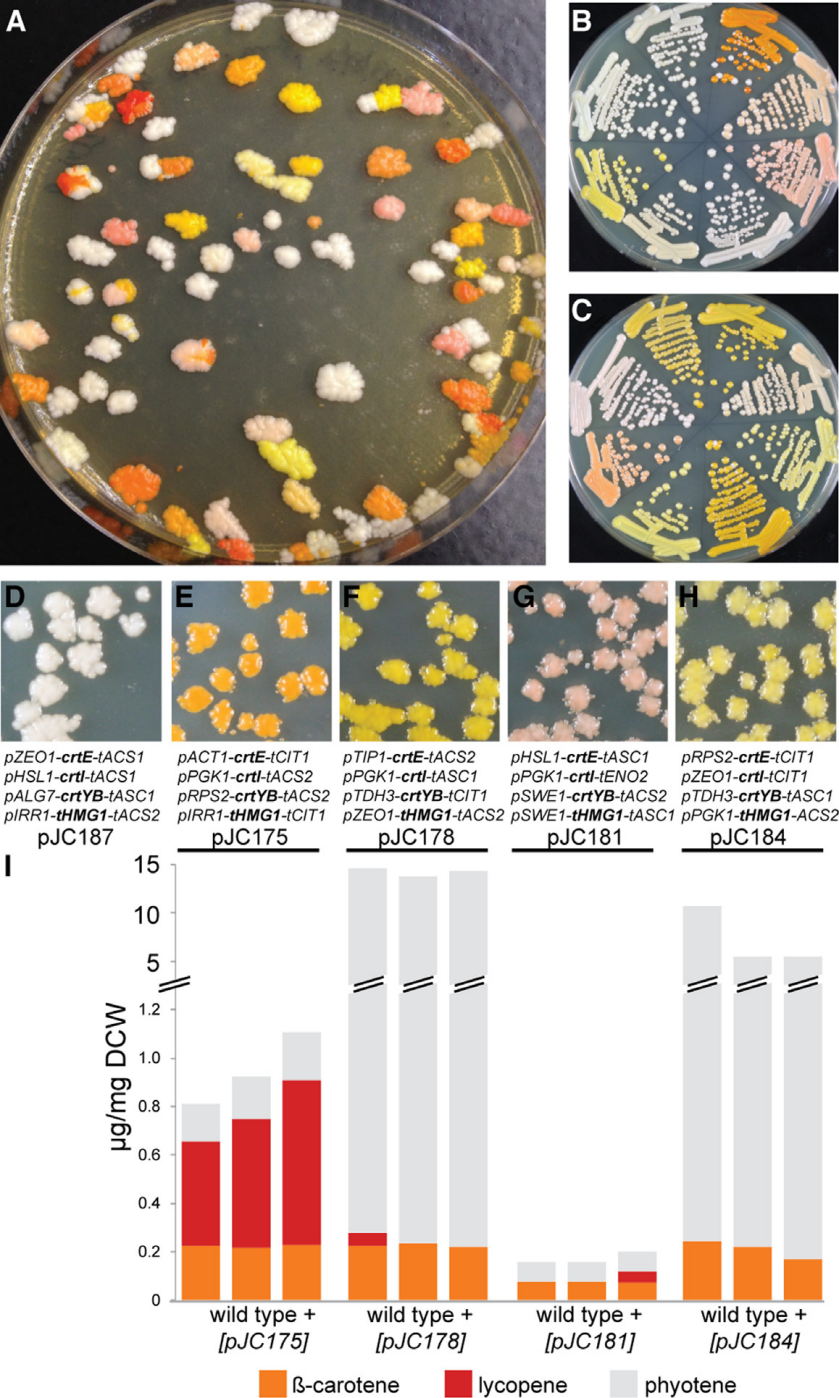
Combinatorial assembly of the β-carotene pathway in *S. cerevisiae*. (**A**) Combinatorial TU libraries of the four β-carotene pathway genes (*crtE, crtI, crtYB* and *tHMG1*) were generated by yGG and assembled for expression in yeast by VEGAS as in Figure 6 except with pools of 10 PRO and 5 TER parts for each yGG assembly of each TU. Transformants of varying colors reflect production of different levels of β-carotene and its intermediates due to varied expression of all genes in the pathway leading to different concentrations of both end product and intermediates. (**B and C**) Single colony purification of transformants in (A). (**D-H**) Five assembled constructs were recovered from yeast into *E. coli* (pJC175, pJC178, pJC181, pJC184 and pJC187) and sequenced to identify the promoters and terminators driving expression of each pathway gene. Each construct was also re-transformed into yeast to verify production of β-carotene (and intermediates) based on the yeast colonies developing color uniformly. Shown are replica plates on YPD supplemented with G418. (**I**) HPLC quantification of carotenoids produced in strains (E–H). In all cases, ∼12.5 g of yeast (dry cell weight (DCW)) was used for the analysis. Quantification was performed in biological triplicate for each strain as shown. All strains analyzed contained additional carotenoid peaks that may have contributed to color formation.

A major advantage of VEGAS is its compatibility with combinatorial assembly, made possible by the modularity provided by the VA sequences. To demonstrate this, using yGG we generated combinatorial TU libraries for each of the four β-carotene pathway genes and then used PCR-mediated VEGAS to assemble the TU libraries into combinatorial pathways for expression in yeast. With a pool of 10 PROs and 5 TERs in each TU combinatorial assembly (Table 5), the theoretical library complexity exceeded 60,000 possible combinations. For this experiment the 5 TUs were assigned the same VAs as in Table 3, so the same primers were used to generate amplicons with terminal homology. Following VEGAS in *S. cerevisiae*, we observed a wide diversity of colony colors on the transformation/G418 replica plate (Figure 7A). We interrogated the stability and robustness of expression of the assembled pathways by re-streaking transformants representing many different colors for single colonies (Figure 7B and C.) Sequence analysis of five constructs conferring uniquely colored yeast colonies (orange, bright yellow, light pink, light yellow and white) revealed the presence of all 10 PROs and 4 of the 5 TERs in at least one position in an assembled pathway, consistent with unbiased combinatorial assembly reactions (Figure 7E–H). Finally, we assessed the production of three carotenoid compounds in yeast cells expressing four unique β-carotene pathways (strains from Figure 7D–G). Indeed, we observed different abundances of β-carotene, phytoene and lycopene in these strains (Figure 7I). While each of the yellow and orange strains produce two to three times more β-carotene than the pink colored strain, it is likely the abundance of lycopene that differentiates the orange from the yellow strain. On the other hand, both yellow strains produce an abundance of phytoene, an early intermediate in the β-carotene pathway (13), suggesting that flux could still be improved by additional pathway engineering; alternatively, additional transformants could be screened to identify assembled pathways that yield higher β-carotene titers.

**Table 5.** Promoter and terminators pools for combinatorial assembly

PRO	TER
*pTDH3*	*ttACS2*
*pPGK1*	*ttENO2*
*pACT1*	*ttASC1*
*pRPS2*	*ttCIT1*
*pZEO1*	*ttSIK1*
*pIRR1*	
*pALG7*	
*pSWE1*	
*pTIP1*	
*pHSL1*	

## DISCUSSION

Biosynthetic pathways typically consist of multiple genes whose individual protein products function much like an assembly line, converting an initial substrate, through some number of intermediate steps, into a desired end product. Expressing biosynthetic pathways in *S. cerevisiae*, in particular those not natively encoded in the *S. cerevisiae* genome, is desirable as it effectively converts this microorganism into a cellular factory capable of producing valuable compounds. A major consideration is tuning expression of individual genes to optimize flux through the pathway, given that balanced gene expression can often trump simple overexpression of each pathway gene with respect to yield. High-level constitutive expression may create a significant metabolic burden on the cell, or lead to the accumulation of toxic foreign intermediates. For example, violacein is toxic to yeast cells at high concentration (21), which may contribute to the slower growth of purple colonies as compared to white ones on the VEGAS violacein assembly plates (Figure 6D).

Here we address the challenge of assembling and tuning genetic pathways with VEGAS, a modular approach that allows facile assembly of TUs flanked by VAs into complete genetic pathways by homologous recombination in yeast. Gene expression can be controlled by assigning desired regulatory elements (PRO and TER parts) up front or, as we demonstrate for the β-carotene pathway, in a combinatorial manner during the yGG reaction. Many previous studies investigating the expression of β-carotene expression in *S. cerevisiae* (22–24) have relied on a previously built construct encoding *crtE, crtI* and *crtYB*, each expressed using an identical PRO and TER combination (13). Here, using VEGAS / yGG we construct and characterize six new β-carotene pathway expression cassettes; in principle we could characterize any number of additional constructs assembled using the combinatorial approach. These constructs represent useful new resources since they display a high degree of genetic stability in yeast, evidenced by the uniformity of colony color (Figures 6 and 7). Presumably the observed genetic stability is a function of the use of unique PROs and TERs flanking each CDS. Notably, the constructs derived from the combinatorial assembly share at least one common part (Figure 7D–H); in future combinatorial assembly experiments, this could easily be overcome by increasing the number of PRO and TER parts used during combinatorial yGG assembly.

VEGAS specifies episomal expression of the assembled genetic pathway, which comes with advantages and disadvantages. Episomal expression allows one to leverage a variety of systematic screening tools available for *S. cerevisiae*, for instance the deletion mutant collection (25) or the overexpression array (26), since the pathway can easily be moved between strains. Moreover, state-of-the-art approaches such as SCRaMbLE (27) of synthetic chromosomes (28,29) constructed as part of the Sc2.0 Synthetic Yeast Genome Project (www.syntheticyeast.org) can be implemented to identify favorable genetic backgrounds for pathway expression. However, the use of selective medium or the addition of a drug to ensure maintenance of the pathway construct may lead to decreased product yield. One simple solution is to make the plasmid essential in the strain background such that it cannot be lost (30); this approach could certainly be implemented either as part of the VEGAS workflow or at a later date once the desired construct is introduced into the most favorable strain background. Of course, a VEGAS assembly vector could also be constructed (or retrofitted) such that following episomal VEGAS pathway assembly and characterization the pathway could be integrated into the genome.

The use of computationally derived orthogonal sequences provides a powerful tool for DNA assembly, as described here using yeast and elsewhere using *in vitro* methods (31,32). *S. cerevisiae*, with its inherent capacity for homologous recombination, is an incredible cloning tool; the standardized and modular assembly of genetic pathways by yGG/VEGAS need not be limited to expression in *S. cerevisiae*. Rather, pathways assembled episomally in yeast using this approach can easily be transferred to other microorganisms, in particular those that are not proficient at homologous recombination. Here, a ‘next generation’ VEGAS vector would require parts for selection, replication and segregation in the destination organism.
